# Grave Injuries to the Abdominal Walls

**Published:** 1899-02-11

**Authors:** 


### GRAVE INJURIES TO THE ABDOMINAL WALLS.

A CASE in which a woman was disembowelled by a cow, reported by Dr. Thomas Bell in the *Lancet* of last week, and another reported by Dr. J. R. Jenkins the week before, in which the small intestines protruded through a wound made in the abdominal walls by a bicycle accident, should encourage one to treat such accidents in full hope of a successful result, and not be led by the apparent imminence of death to omit any precaution which may conduce to recovery. In the first case the wound was six inches in length, and through it 18 inches of the transverse colon and the whole of the mesenteric portion of the jejunum and ileum were protruding—about 20 feet of the small bowel. The wound was washed with 1 in 20 carbolic, and the bowels with 1 in 40, and after the bowels had been reduced the wound was closed. From the time of the goring to the end of the reduction about four hours elapsed. The patient recovered. In the second case the shaft on the off-side of a passing cart struck the cyclist on the right thigh, and, gliding upwards over the groin, penetrated the abdominal wall, tearing this open to the extent of about seven inches. There was immediate protrusion of bowel. When he was seen it was found that six or seven coils of intestine were protruded to the extent of five inches. They were, however, uninjured. Reduction was difficult, and could only be effected after making a transverse incision. After "due antiseptic treatment" the wound was closed and dressed with iodoform and cotton wool. The patient recovered.

